# Supplementary material for: Coevolution of Codependent Hosts and Symbionts
Source: bioRxiv. 2026 Jul 24:2026.07.21.739856. Preprint. [Version 1] doi: 10.64898/2026.07.21.739856 (PMC13420449; doi:10.64898/2026.07.21.739856)
Supplement: 1 [file NIHPP2026.07.21.739856v1-supplement-1.pdf]

bioRxiv preprint doi: https://doi.org/10.64898/2026.07.21.739856; this version posted July 24, 2026. The copyright holder for this preprint (which was not certified by peer review) is the author/funder, who has granted bioRxiv a license to display the preprint in perpetuity. It is made available under a CC-BY 4.0 International license.

SUPPLEMENTAL TEXT

A) Situation in which the symbiont trait only influences symbiont fitness

In this case, the host trait evolves towards its upper limit, to a degree that only depends on the scaled selection coefficient δN and the host mutation bias b_H. As the relative fitnesses of alternative symbiont types depend only on the number of + alleles in their genotype (here denoted as j), and not on the host-cell background, the symbiont fitness can be written as

$$W_S(j) = e^{-\beta j}, \tag{A1}$$

so that the selective difference between any two adjacent trait values is β, with reductions in the index j being advantageous. We further assume that symbiont mutation rates and population sizes are independent of j.

Here, we consider the transition probabilities (U) between adjacent symbiont states within individual host cells, which are functions of the mutation rates to destination alleles and their probabilities of fixation. For changes to the next highest index,

$$\begin{aligned} U_{j\to j+1} &= b_S u_S K(L_s - j)\left(\frac{1-e^{2\beta}}{1-e^{2K\beta}}\right) \\ &\simeq b_S u_S (L_s - j) K \left(\frac{2\beta}{e^{2K\beta}-1}\right), \end{aligned} \tag{A2a}$$

where the final approximation holds for $\beta \ll 1$. For changes to the next lowest index,

$$\begin{aligned} U_{j\to j-1} &\simeq u_S j K \left(\frac{2\beta}{1-e^{-2K\beta}}\right) \\ &= u_S j K \left(\frac{2\beta}{e^{2K\beta}-1}\right) e^{2K\beta} \end{aligned} \tag{A2b}$$

where L_s is the number of genomic sites, K is the number of symbionts per host cell, u_S is the mutation rate from + to − alleles, and $b_S u_S$ is the reciprocal mutation rate. Thus, these two transition types are equivalent to an effective mutation process with forward rate

$$\mu = b_S u_S K \left(\frac{2\beta}{e^{2K\beta}-1}\right), \tag{A3a}$$

and backward rate μB with

$$B = e^{2K\beta}/b_S \tag{A3b}$$

bioRxiv preprint doi: https://doi.org/10.64898/2026.07.21.739856; this version posted July 24, 2026. The copyright holder for this preprint (which was not certified by peer review) is the author/funder, who has granted bioRxiv a license to display the preprint in perpetuity. It is made available under a CC-BY 4.0 International license.

denoting the directional bias. Finally, the rate at which a host cell changes from state j to k by migration (assumed to be a single immigrant per host cell per migration event) is

$$m_{j\to k}p_k \simeq mp_k \left(\frac{2\beta(k-j)}{e^{2K\beta(k-j)}-1} \right), \tag{A4}$$

assuming $\beta L_s \ll 1$, where m is the frequency of migration events per host cell, and p_k is the frequency of type k symbionts across the entire host population (and in the pool of migrants).

Assuming that joint mutation / migration events do not occur, the general recursion equation for the haplotype frequencies of symbionts (assumed to be homoplasmic) is

$$\begin{aligned} p'_j = &\left(1 - U_{j\to j+1} - U_{j\to j-1} - \sum_{j\neq k} m_{j\to k}p_k\right) p_j \\ &+ U_{j-1\to j}p_{j-1} + U_{j+1\to j}p_{j+1} + \sum_{j\neq k} m_{k\to j}p_j p_k, \end{aligned} \tag{A6a}$$

assuming all U and m terms are $\ll 1$, which rearranges to

$$\begin{aligned} p'_j = &(1 - U_{j\to j+1} - U_{j\to j-1})p_j + U_{j-1\to j}p_{j-1} + U_{j+1\to j}p_{j+1} \\ &+ \sum_{j\neq k}(m_{k\to j} - m_{j\to k})p_j p_k, \end{aligned} \tag{A6b}$$

where the two rows, respectively, denote changes due to mutation and migration. Using Equation A4, the migration term simplifies further to

$$\begin{aligned} m_{k\to j} - m_{j\to k} &= m\left(\frac{2\beta(j-k)}{e^{2K\beta(j-k)}-1} - \frac{2\beta(k-j)}{e^{2K\beta(k-j)}-1} \right) \\ &= m\left(\frac{2\beta(j-k)}{e^{2K\beta(j-k)}-1} \right)(1 - e^{2K\beta(j-k)}) \\ &= 2m\beta(k-j), \end{aligned} \tag{A7}$$

which should be a good approximation provided $\beta L_s \ll 1$. With this transformation, state j can be viewed as having an absolute selection coefficient equal to $-2m\beta j$ relative to the benchmark of zero, such that the selective differences between adjacent states are $2m\beta$ for any $j \to j-1$ and $-2m\beta$ for any $j \to j+1$.

Approximation for the small-population-size domain. The preceding analysis reveal a rescaled system of equations with constant effective rates of forward and reverse terms for the

bioRxiv preprint doi: https://doi.org/10.64898/2026.07.21.739856; this version posted July 24, 2026. The copyright holder for this preprint (which was not certified by peer review) is the author/funder, who has granted bioRxiv a license to display the preprint in perpetuity. It is made available under a CC-BY 4.0 International license.

combined effects of mutation and within-host selection per site and linear dependence of the fitness differences between haplotypes and their individual states. In the small-population-size regime, the population remains largely monomorphic with rare excursions to adjacent states, initiated by a host cell containing a one-step change in its endosymbionts caused by mutation and within-host selection, with such a variant then having the capacity to spread through the entire host population via the joint process of migratory exchange and within-host selection.

This process has the same form as that for a linear array of nonrecombining haplotypes with L_S biallelic $(+/-)$ sites, with constant rates of mutation per site type and constant strengths of selection between adjacent haplotypes. The balance between the joint forces of mutation, selection, drift, and migration leads to predictions for the steady-state distribution of alternative haplotypes, where j indeses the number of $+$ alleles,

$$\begin{aligned}\widetilde{P}_j &= C \cdot \binom{L_S}{j} B^{-j} e^{-2N(2m\beta j)} \\ &= C \cdot \binom{L_S}{j} b_S^j \cdot e^{-2\beta(2mN+K)j}, \qquad \text{(A8)}\end{aligned}$$

where $C = [1 + b_S e^{-2\beta(2Nm+K)}]^{-L_S}$ is the normalization constant needed to ensure that the frequencies sum to 1.0 (Foundations 14.2; Lynch 2024).

In this small-population-size domain and with the exponential fitness function, the mean frequency of $+$ alleles (equivalent to the fraction of time spent as $+$) is unaffected by selective modification at other sites, and is given by the standard Li-Bulmer equation,

$$\widetilde{p}_+ = \frac{b_S}{b_S + e^{2\beta(2Nm+K)}} \qquad \text{(A9)}$$

(Li 1987; Bulmer 1991). Equation A9 shows that the total number of migration events per generation (mN) boosts the effective population size of the symbiont by 2 (for unclear reasons....) The mean number of $+$ alleles in the haplotypic array is $\widetilde{p}_+ L_S$, and the mean symbiont state (on the scale of $-E_S$ to $+E_S$) is

$$\overline{s} = -E_S(1 - 2\widetilde{p}_+). \qquad \text{(A10)}$$

Approximation for the large-population-size domain. Here, we assume a host population that is effectively infinite in size, which under the assumption of linkage equilibrium, allows the solution to the deterministic equation

$$-2m\beta\widetilde{p}_+(1 - \widetilde{p}_+) + \mu(1 - \widetilde{p}_+) - B\mu\widetilde{p}_+ = 0$$

bioRxiv preprint doi: https://doi.org/10.64898/2026.07.21.739856; this version posted July 24, 2026. The copyright holder for this preprint (which was not certified by peer review) is the author/funder, who has granted bioRxiv a license to display the preprint in perpetuity. It is made available under a CC-BY 4.0 International license.

which rearranges to

$$\widetilde{p}_{+} = \frac{2m\beta + (1+B)\mu - \sqrt{[2m\beta + (1+B)\mu]^2 - 8m\beta\mu}}{4m\beta}. \tag{A11a}$$

If either $m \ll$ or $m \gg u_s K(1+e^{-2\beta K})/(1-e^{-2\beta K})$ or $m \ll u_s K(1+e^{-2\beta K})/(1-e^{-2\beta K})$, then

$$\widetilde{p}_{+} \simeq \frac{u_s K}{(u_s K + m)e^{2\beta K} + u_s K - m}. \tag{A11b}$$

B) Situation in which the symbiont trait also influences symbiont population size

Here, we attempt to generalize the preceding results to allow for the possibility that symbiont genotypes also influence the within-host population size in a frequency-dependent manner. Letting the population sizes of pure symbiont populations be

$$K_i = \frac{\kappa}{1 - \lambda_S[i - (L_S/2)]}, \tag{B1}$$

where i is the number of + alleles within the haplotype of length $2E_s$, the weighting factor $|\lambda_S|$ must be $< 1/E_S$, and κ is the benchmark symbiont population size when the haplotype is at the midpoint on the symbiont genotype array ($i = L_s/2$). For $|\lambda_S| \ll 1/E_S$, $K_j \simeq \kappa e^{\lambda_S(2j-E_S)}$. Although other functions for K_i can be imagined, this particular function proves useful mathematically in the derivations below.

A fairly general model for the response of the total intrahost population size for a mixed pair of symbionts of types j and k, with respective frequencies p_j and p_k (which sum to 1.0, under the assumption of no more than two symbiont types per host cell at any point in time) is given by

$$K(i,j) = [\, K_i^{\gamma} p_i + K_j^{\gamma} p_j \,]^{1/\gamma} \tag{B2}$$

(Joshi et al. 2027). If $\gamma = 1$, K is simply the weighted arithmetic mean of the two haplotype-specific population sizes, whereas $\gamma = -1$ implies a harmonic mean, $\gamma = 2$ is a root mean-squared model, and $\gamma \to 0$ yields a geometric mean. This function is required to determine the fixation probabilities of newly arisen mutations of type j invading a type i population, and changing its frequency in doing so, and vice versa.

An approximate solution to the steady-state distribution of symbiont haplotypes under the small-population-size regime, where transitions between states occur by single-step mutations

bioRxiv preprint doi: https://doi.org/10.64898/2026.07.21.739856; this version posted July 24, 2026. The copyright holder for this preprint (which was not certified by peer review) is the author/funder, who has granted bioRxiv a license to display the preprint in perpetuity. It is made available under a CC-BY 4.0 International license.

only, can be obtained by noting that under detailed balance for all adjacent pairs of haplotypes,

$$\widetilde{p}_i \cdot K_i \cdot \mu_{i \to i+1} \cdot \phi_{i \to i+1} = \widetilde{p}_{i+1} \cdot K_{i+1} \cdot \mu_{i+1 \to i} \cdot \phi_{i+1 \to i}, \tag{B3}$$

where $\mu_{i \to i+1}$ and $\mu_{i+1 \to i}$ denote forward and reverse mutation rates, and $\phi_{i \to i+1}$ and $\phi_{i+1 \to i}$ are the fixation probabilities for forward and reverse mutations. The solution to the full set of equations requires expressions for the ratios of fixation probabilities $\phi_{i \to i+1}/\phi_{i+1 \to i}$, which have the simple form of $e^{2K\beta}$ (see above) when K is constant, but are otherwise more complicated. Although a general expression for the fixation-probability formula has been obtained for the case of frequency-dependent change in K (Equation 3a in Joshi et al. 2027), even the expressions for special cases are complicated and nontransparent, as they contain terms in the form of gamma functions, error functions, and exponential integral functions.

Harmonic-mean model. Some progress is possible for the case in which $\gamma = -1$,

$$K(i,j) = \left(\frac{p_i}{K_i} + \frac{p_j}{K_j} \right)^{-1}, \tag{B4}$$

where $p_i + p_j = 1$, and in the excursion of any mutant allele, the allele frequencies are stochastic variables en route to allele loss or fixation, and

$$\phi_{i \to i+1} = \frac{\{\, 1 + (1/K_{i+1}) - (1/K_i) \,\}^x - 1}{[K_i/K_{i+1}]^x - 1}, \tag{B5a}$$

is the probability of fixation of haplotype $i+1$ on an i background (for which there is a selective disadvantage of $i+1$ relative to i of β), with

$$x = 1 + 2\beta K_i K_{i+1}/(K_i - K_{i+1}), \tag{B5b}$$

and $\phi_{i+1 \to i}$ obtained by reversing the subscripts in Equation B4, and changing the sign of β.

Assuming K_i, $K_{i+1} \gg 1$ and using $(1 + 1/K)^a \simeq 1 + (a/K)$, leads to

$$\frac{K_i \phi_{i \to i+1}}{K_{i+1} \phi_{i+1 \to i}} \simeq \left(\frac{K_{i+1}}{K_i} \right)^{x-1}. \tag{B6a}$$

Using Equation B1 yields the recursion equation,

$$\frac{1}{K_{i+1}} = \frac{1}{K_i} - \frac{\lambda_S}{\kappa},$$

bioRxiv preprint doi: https://doi.org/10.64898/2026.07.21.739856; this version posted July 24, 2026. The copyright holder for this preprint (which was not certified by peer review) is the author/funder, who has granted bioRxiv a license to display the preprint in perpetuity. It is made available under a CC-BY 4.0 International license.

reducing things further to

$$\frac{K_i \phi_{i\to i+1}}{K_{i+1}\phi_{i+1\to i}} \simeq \left(\frac{K_{i+1}}{K_i}\right)^{-2\beta\kappa/\lambda_S}. \tag{B6b}$$

Returning to Equation B3, and noting that $\mu_{i\to i+1}/\mu_{i+1\to i} = (L_S - i)b_S/(i+1)$, where L_S is the haplotype length, we obtain

$$\widetilde{p}_{i+1} = \widetilde{p}_i \cdot \frac{(L_S - i)b_S}{i+1} \cdot \left(\frac{K_i}{K_{i+1}}\right)^{2\beta\kappa/\lambda_S}, \tag{B7a}$$

which after applying Equation B1 implies

$$\widetilde{p}_1 = \widetilde{p}_0 \cdot (L_S b_S) \cdot \left[\frac{1 + \lambda_S(L_S/2 - 1)}{1 + \lambda_S L_s/2}\right]^{2\beta\kappa/\lambda_S}, \tag{B7b}$$

and recursion leads to the general formula

$$\widetilde{p}_i = C \cdot \binom{L_S}{i} b_S^i([1 - \lambda_S(i - L_s/2)]^{2\beta\kappa/\lambda_S}, \tag{B7c}$$

where C is the normalization constant that ensures that the frequencies sum to 1.0. In the limit, as $\lambda_S \to 0$ (a constant population size K), the term to the right converges on $\exp[-2\beta K(i - L_S/2)]$, and absorbing the constant part containing $L_S/2$ into the normalization constant yields

$$\widetilde{p}_i = C \cdot \binom{L_S}{i} b_S^i e^{-2\beta K i}, \tag{B7d}$$

the standard expression for a linear array of alleles separated by constant strength of selection in a population of constant size (Equation A8). Equations B7c,d neatly separate the steady-state distribution into components due to mutation alone (the binomial neutral expectation) and its modification by selection.

Equation B7b assumes strict vertical inheritance of symbionts, but can be generalized to include migration in the following way. Using the same approach as in Equation A7, but with fixation probabilities appropriate for the harmonic-mean value, and assuming K_i, $K_j \gg 1$, after some algebra,

$$m_{j\to i} - m_{i\to j} \simeq m\left(\frac{1}{K_j} - \frac{1}{K_i} - 2\beta(i-j)\right), \tag{B8}$$

leading to the conclusion that migration leads haplotype i to have a net selective effect of

$$m_i = -m\left(2\beta i + \frac{1}{K_i}\right) \tag{B9a}$$

bioRxiv preprint doi: https://doi.org/10.64898/2026.07.21.739856; this version posted July 24, 2026. The copyright holder for this preprint (which was not certified by peer review) is the author/funder, who has granted bioRxiv a license to display the preprint in perpetuity. It is made available under a CC-BY 4.0 International license.

with application of Equation B1 leading to

$$m_i = -mi\left(2\beta - \frac{\lambda_S}{\kappa}\right). \tag{B9b}$$

Equation B7c can then be expanded to include the selective effects associated with migration

$$\widetilde{p}_i = C \cdot \binom{L_S}{i} b_S^i \cdot [1 - \lambda_S(i - L_S/2)]^{2\beta\kappa/\lambda_S} \cdot e^{2Ni[\alpha - 2m\beta + (m\lambda_S/\kappa)]}, \tag{B10}$$

where we have included the term to account for selection at the host-cell level (here assuming that host population size N remains constant). Except for the inclusion of selection at the host level, reduces to Equation A8 with constant symbiont population size.

Finally, if the host population size is influenced by the symbiont haplotype, using a function of similar form for the symbiont population size,

$$N_i = \frac{\eta}{1 - \lambda_H[i - (L_S/2)]}, \tag{B11}$$

similar steps lead to

$$\widetilde{p}_i = C \cdot \binom{L_S}{i} b_S^i \cdot ([1 - \lambda_S(i - L_S/2)]^{2\beta\kappa/\lambda_S} \cdot [1 - \lambda_H(i - L_s/2)]^{-(2\eta/\lambda_H)[\alpha - 2m\beta + (m\lambda_S/\kappa)]}. \tag{B12}$$

C) Limits on the domain for the sequential model

The sequential-fixation regime can be approximated by requiring the time to fixation to be less than the time to arrival of next mutation destined to fix. Because the time to arrival of a deleterious mutation destined to fix is generally much greater than that of a beneficial mutation (except in the neutral regime), we focus on the time to arrival of beneficial mutations destined to fix. Under sufficiently strong selection ($Ns_b \gg 1$), where s_b is the selective advantage of the beneficial relative to the deleterious allele, the time to fixation $\simeq 2\ln(N)/s_b$, and the probability of fixation $\simeq 2s_b$. Thus, the condition for sequential fixation is,

$$\frac{2\ln N}{s_b} < \frac{1}{2s_b N L_d \mu_{d\to b}},$$

where L_d is the average number of sites with deleterious mutations in steady state, and $\mu_{d\to b}$ is the mutation rate per site from the deleterious to beneficial state. Rearranging,

$$4L_d\mu_{d\to b} N \ln N < 1. \tag{C1}$$

bioRxiv preprint doi: https://doi.org/10.64898/2026.07.21.739856; this version posted July 24, 2026. The copyright holder for this preprint (which was not certified by peer review) is the author/funder, who has granted bioRxiv a license to display the preprint in perpetuity. It is made available under a CC-BY 4.0 International license.

Application to two levels of selection. In the system studied here, for the host population, $s_b = \delta$, and L_d can be found from the Li-Bulmer solution,

$$L_d = L_h\left(1 - \frac{b_h e^{2N\delta}}{b_h e^{2N\delta} + 1}\right) = \frac{L_h}{b_h e^{2N\delta} + 1}$$

Thus, the condition is,

$$\frac{4 b_h u_h L_h N \ln N}{1 + b_h e^{2N\delta}} < 1. \tag{C2}$$

For the symbiont population, following from the text, the net selection coefficient, $s' = \alpha - 2m\beta$, can be positive or negative, and the identity of the beneficial mutation changes accordingly. The mutation rate towards beneficial mutation also changes,

$$\begin{aligned} \mu_{d\to b} &= b_s u_s \frac{2K\beta}{e^{2K\beta} - 1} && \text{for } \alpha > 2m\beta, \\ &= u_s \frac{2K\beta}{1 - e^{-2K\beta}} && \text{for } \alpha < 2m\beta. \end{aligned}$$

The expected number of sites with deleterious mutations is now,

$$\begin{aligned} L_d &= L_s\left(1 - \frac{b_s}{b_s + e^{2\beta K - 2(\alpha - 2m\beta)N}}\right) && \text{for } \alpha > 2m\beta, \\ &= \frac{b_s L_s}{b_s + e^{2\beta K - 2(\alpha - 2m\beta)N}} && \text{for } \alpha < 2m\beta. \end{aligned}$$

Thus, the conditions necessary to satisfy the sequential model become,

$$\frac{8K\beta L_s b_s u_s N \ln N}{\epsilon} < 1, \tag{C3a}$$

where,

$$\begin{aligned} \epsilon &= (1 + b_s e^{2(\alpha - 2m\beta)N - 2\beta K})(e^{2\beta K} - 1) && \text{for } \alpha > 2m\beta, \\ &= (b_s + e^{2\beta K - 2(\alpha - 2m\beta)N})(1 - e^{-2\beta K}) && \text{for } \alpha < 2m\beta. \end{aligned}$$

Because we require an overall situation where sequential fixation applies to both host and symbiont traits, we require that Equations 2 and 3 hold simultaneously.

Literature Cited

Bulmer, M. 1991. The selection-mutation-drift theory of synonymous codon usage. Genetics 129: 897-907.

bioRxiv preprint doi: https://doi.org/10.64898/2026.07.21.739856; this version posted July 24, 2026. The copyright holder for this preprint (which was not certified by peer review) is the author/funder, who has granted bioRxiv a license to display the preprint in perpetuity. It is made available under a CC-BY 4.0 International license.

Joshi, K., Halder, S., A. González Casanova, and M. Lynch. 2027. Fixation probabilities of mutant alleles in an ecological context. (manuscript under review).

Li, W. H. 1987. Models of nearly neutral mutations with particular implications for non-random usage of synonymous codons. J. Mol. Evol. 24: 337-345.

Lynch, M. 2024. Evolutionary Cell Biology: The Origins of Cellular Architecture. Oxford Univ. Press, Oxford, UK.
